# Extracellular vesicle dynamics in COPD: understanding the role of miR-422a, SPP1 and IL-17 A in smoking-related pathology

**DOI:** 10.1186/s12890-024-02978-y

**Published:** 2024-04-12

**Authors:** Zhihui Dai, Li Lin, Yanan Xu, Lifang Hu, Shiping Gou, Xinkai Xu

**Affiliations:** Department of Respiratory and Critical Care Medicine, Yongkang First People’s Hospital, Hangzhou Medical College, No. 599 Jinshan West Road, 321300 Yongkang, Zhejiang Province P. R. China

**Keywords:** COPD, Extracellular vesicles, miR-422a, SPP1, IL-17 signaling pathway, Bioinformatics

## Abstract

**Background:**

Chronic obstructive pulmonary disease (COPD) induced by smoking poses a significant global health challenge. Recent findings highlight the crucial role of extracellular vesicles (EVs) in mediating miRNA regulatory networks across various diseases. This study utilizes the GEO database to uncover distinct expression patterns of miRNAs and mRNAs, offering a comprehensive understanding of the pathogenesis of smoking-induced COPD. This study aims to investigate the mechanisms by which extracellular vesicles (EVs) mediate the molecular network of miR-422a-SPP1 to delay the onset of COPD caused by smoking.

**Methods:**

The smoking-related miRNA chip GSE38974-GPL7723 was obtained from the GEO database, and candidate miRs were retrieved from the Vesiclepedia database. Downstream target genes of the candidate miRs were predicted using mRNA chip GSE38974-GPL4133, TargetScan, miRWalk, and RNA22 databases. This prediction was integrated with COPD-related genes from the GeneCards database, downstream target genes predicted by online databases, and key genes identified in the core module of WGCNA analysis to obtain candidate genes. The candidate genes were subjected to KEGG functional enrichment analysis using the “clusterProfiler” package in R language, and a protein interaction network was constructed. In vitro experiments involved overexpressing miRNA or extracting extracellular vesicles from bronchial epithelial cell-derived exosomes, co-culturing them with myofibroblasts to observe changes in the expression levels of the miR-422a-SPP1-IL-17 A regulatory network, and assessing protein levels of fibroblast differentiation-related factors α-SMA and collagen I using Western blot analysis.

**Results:**

The differential gene analysis of chip GSE38974-GPL7723 and the retrieval results from the Vesiclepedia database identified candidate miRs, specifically miR-422a. Subsequently, an intersection was taken among the prediction results from TargetScan, miRWalk, and RNA22 databases, the COPD-related gene retrieval results from GeneCards database, the WGCNA analysis results of chip GSE38974-GPL4133, and the differential gene analysis results. This intersection, combined with KEGG functional enrichment analysis, and protein-protein interaction analysis, led to the final screening of the target gene SPP1 and its upstream regulatory gene miR-422a. KEGG functional enrichment analysis of mRNAs correlated with SPP1 revealed the IL-17 signaling pathway involved. In vitro experiments demonstrated that miR-422a inhibition targets suppressed the expression of SPP1 in myofibroblasts, inhibiting differentiation phenotype. Bronchial epithelial cells, under cigarette smoke extract (CSE) stress, could compensate for myofibroblast differentiation phenotype by altering the content of miR-422a in their Extracellular Vesicles (EVs).

**Conclusion:**

The differential gene analysis of Chip GSE38974-GPL7723 and the retrieval results from the Vesiclepedia database identified candidate miRs, specifically miR-422a. Further analysis involved the intersection of predictions from TargetScan, miRWalk, and RNA22 databases, gene search on COPD-related genes from the GeneCards database, WGCNA analysis from Chip GSE38974-GPL4133, and differential gene analysis, combined with KEGG functional enrichment analysis and protein interaction analysis. Ultimately, the target gene SPP1 and its upstream regulatory gene miR-422a were selected. KEGG functional enrichment analysis on mRNAs correlated with SPP1 revealed the involvement of the IL-17 signaling pathway. In vitro experiments showed that miR-422a targeted inhibition suppressed the expression of SPP1 in myofibroblast cells, inhibiting differentiation phenotype. Furthermore, bronchial epithelial cells could compensate for myofibroblast differentiation phenotype under cigarette smoke extract (CSE) stress by altering the miR-422a content in their extracellular vesicles (EVs).

**Supplementary Information:**

The online version contains supplementary material available at 10.1186/s12890-024-02978-y.

## Background

Chronic Obstructive Pulmonary Disease (COPD) stands out as a debilitating respiratory condition marked by its progressive nature and the partial irreversibility of its airflow constraints [1]. As statistics reveal, COPD is swiftly rising as a dominant cause of mortality worldwide. Among the various environmental triggers, tobacco smoking is unmistakably the most potent factor precipitating COPD’s development [2]. The chronic inhalation of smoke triggers a cascade of pathological alterations in the respiratory system, including persistent airway inflammation, bronchial narrowing, and subsequent airflow limitation, culminating in a pronounced decline in both the quality and span of the patient’s life [3].

Extracellular vesicles (EVs) are heterogeneous nanoscale membrane vesicles released by cells for intercellular communication and are surrounded by a double lipid bilayer that protects them from degradation [4]. Multivesicular bodies (MVBs) and intraluminal vesicles (ILVs) within the MVBs are formed through several processes that contribute to EV generation [5]. Initially, early-sorting endosomes (ESEs) are formed due to invagination of the plasma membrane [6]. ESEs then evolve into late-sorting endosomes (LSEs), which ultimately generate MVBs containing ILVs through inward budding of the limiting membrane [7–9]. Finally, ILVs are released into the extracellular environment through the fusion of MVBs with the plasma membrane, confirming the endosomal pathway of exosome formation [8, 10]. The intraluminal vesicle formation model of exosome biogenesis is supported by extensive data from genetic and electron microscopy studies [11]. EVs are classified into three main subgroups based on their biological development and origin: exosomes, microvesicles, and apoptotic bodies [12], all playing crucial roles in various physiological and pathological processes, including intercellular communication, immune regulation, and tissue repair [13]. Although the origin of these EVs is understood, the current techniques for isolation and characterization are unable to clearly distinguish between different types of EVs, leading to significant naming confusion [14, 15]. Therefore, this study uses the term EVs universally. As self-secreted/paracrine effectors, EVs transport metabolic products, proteins, and nucleic acids, including mRNA and miRNA. Among them, miRNA is considered a crucial factor in their functional role. miRNAs are a class of non-coding small RNA molecules that regulate the translation of target mRNAs and subsequently influence the physiological functions of cells [16].

In the development of COPD, there is an imbalance between the damage and repair of bronchial epithelial cells, as well as the accumulation of extracellular matrix, leading to remodeling of the bronchial and alveolar structures, ultimately exacerbating airflow limitation [17]. Moreover, immune reactions and changes in the microenvironment play a crucial role in the progression and development of COPD [17]. The airway remodeling, characterized by the differentiation of fibroblasts into myofibroblasts, is a direct result of the inflammatory response induced by cigarette smoke [18, 19]. In recent years, miR-422a has gained wide attention due to its tumor-suppressive function in various types of cancer research. Additionally, it has also shown significant involvement in diseases related to inflammation and fibrosis, such as atherosclerosis involving monocyte recruitment fate [20], macrophage-associated non-alcoholic fatty liver disease (NAFLD) [21], and immune-related lupus nephritis (LN). However, studies on the role of miR-422a in COPD, which is closely associated with inflammation and fibroblast differentiation, remain scarce. SPP1, a secreted phosphoprotein, is located within a quantitative trait locus associated with lung function and is a determinant in murine lung development [22]. Previous research has demonstrated its high expression in CSE-stimulated 16HBE cells [23], and elevated expression of SPP1 in COPD patients is strongly associated with an increased risk of cancer [24]. At a molecular level, SPP1 in COPD can regulate the expression of inflammatory factors through the mediation of the PI3K/Akt signaling pathway [25], but its role in the subsequent airway remodeling during the inflammatory response in COPD remains unclear.

Considering this comprehensive background, our research endeavors to elucidate the intricate dynamics between miR-422a present in the EVs derived from bronchial epithelial cells and its regulatory interplay with SPP1. Through this exploration, we aim to shed light on the underlying molecular mechanisms contributing to the genesis and advancement of COPD instigated by smoking. We optimistically anticipate that this understanding will pave the way for innovative preventive and therapeutic interventions, potentially easing the substantial public health challenge that COPD represents.

## Materials and methods

### Database query

#### GEO chip data retrieval and differential gene analysis

Via the GEO database (https://www.ncbi.nlm.nih.gov/gds), we obtained miRNA microarray GSE38974-GPL7723 related to smoking-induced COPD, which includes 8 smoking healthy controls and 19 smoking COPD patients, and mRNA microarray GSE38974-GPL4133, which encompasses 9 smoking healthy controls and 23 smoking COPD patients.

Using the “limma” package in R, we performed differential gene analysis and plotted volcano plots. The selection criteria were set to|logFC| > 1 and significance *P* < 0.05 to identify differentially expressed mRNAs and miRNAs. Subsequently, we employed the “heatmap” package in R to plot the heatmap of differentially expressed genes.

#### miRNAs candidate selection

In order to accurately predict and screen downstream target genes of candidate miRs, we utilized the TargetScan, miRWalk, and RNA22 databases in conjunction to predict the downstream target genes of candidate miRs [26, 27]. Concurrently, we searched for COPD-related genes in the GeneCards database [28]. To identify genes related to COPD, we conducted a Weighted Gene Co-expression Network Analysis (WGCNA) on the GSE38974-GPL4133 chip data using the R programming language package for WGCNA [29]. Finally, we employed the jvenn tool to intersect the COPD-related genes from the GeneCards database, differentially expressed mRNAs from the GSE38974-GPL4133 chip data, predicted downstream target genes, and key genes from the core module in the WGCNA analysis to obtain candidate mRNAs.

#### Construction of protein-protein interaction (PPI) network

The interaction relationships among candidate mRNAs were analyzed using the STRING database (https://cn.string-db.org/), with the species set to humans. The PPI network was then visualized using the Cytoscape software (version 3.8.2).

#### Pearson correlation analysis and KEGG functional enrichment analysis

The Pearson correlation coefficient was employed to analyze the correlation between differentially expressed mRNAs in the GSE38974-GPL4133 microarray, with a significance threshold set at *P* < 0.05. Using the “clusterProfiler” package in R, KEGG functional enrichment analysis was performed on mRNAs related to SPP1, aiming to discern the primary effects of potential and key targets on cellular functions and signaling pathways. A significance level of *P* < 0.05 was deemed statistically significant.

### In vitro cell culture, treatment, and detection

#### Preparation of cigarette smoke extract (CSE)

The smoke of 3R4F research cigarettes was introduced into a flask containing 10 mL of Minimum Essential Medium (MEM)at a constant rate using a vacuum pump (each cigarette was puffed for 5 min). The pH of the CSE solution was adjusted to 7.4 and then sterile-filtered through a 0.22 μm pore filter (SLGP033N, Sigma-Aldrich, Germany). The solution was standardized for quality control by monitoring absorbance at 320 nm (A320) and 540 nm (A540). If the ΔOD (A320-A540) ranged between 0.9 and 1.2, the quality of the CSE was deemed satisfactory. The resultant CSE solution was considered 100% CSE and was diluted with culture medium for experimental use within 1 h [30].

#### Cell culture

Human bronchial epithelial (HBE) cells (iCell-h321, obtained from Saibai Kang Biological Technology Co., Ltd., China), which are SV40-transformed normal HBE cell lines, and fetal lung fibroblast (MRC-5) cells (CL0161, obtained from Wuhan Punuosi Life Science and Technology Co., Ltd., China) were maintained in minimum essential medium (MEM) supplemented with 10% fetal bovine serum (FBS) (Thermo Fisher Scientific, USA), 100 mg/mL streptomycin, and 100 U/mL penicillin (Thermo Fisher Scientific, USA) at 37 °C under 5% CO_2_. The cells were passaged at a 1:3 ratio every 2 days. Once reaching a confluency of 70–80%, HBE cells were washed with PBS and then grown in MEM supplemented with 10% FBS (the medium was centrifuged overnight at 100,000 g to remove extracellular vesicles) and exposed to 2.5% cigarette smoke extract (CSE) for 12 h to generate extracellular vesicles. In the co-culture model, MRC-5 cells were seeded in 6-well plates and pre-treated in MEM medium containing 10% FBS and 2.5% CSE for 12 h to remove extracellular vesicles. Subsequently, the isolated extracellular vesicles were co-cultured with MRC-5 cells for 24 h.

#### Extracellular vesicle isolation and identification

EVs were isolated from the conditioned medium of either untreated or CSE-treated HBE cells. The conditioned medium was first centrifuged at 3000 g for 15 min, and the supernatant was filtered through a 0.22 μm PVDF filter (Millipore). ExoQuick-TC EV precipitation solution (System Biosciences) was added to the filtered medium in a 1:5 ratio. After mixing and refrigeration for at least 12 h, it was centrifuged at 1500 g for 30 min. For serum EV isolation, 63 µL of ExoQuick EV precipitation solution (System Biosciences) was added to 250 µL of serum. After freezing overnight, the mixture was centrifuged at 1500 g for 30 min. EV pellets were resuspended in 1×PBS. The size distribution and concentration of the EVs were analyzed using a ZetaView particle tracker (ParticleMetrix, Germany).

#### EV uptake assay

EVs derived from HBE cells were labeled with the PKH67 green fluorescent cell linker kit (PKH67GL, Sigma-Aldrich, Germany) following the manufacturer’s protocol with slight modifications. Briefly, EVs suspended in 250 µL of PBS were mixed with 750 µL of a 1:50 dilution of PKH67 (used for membrane labeling, diluted in diluent C) and incubated at room temperature for 5 min. The labeling was quenched by adding 2 ml of 1% BSA. Labeled EVs were isolated, resuspended in 1×PBS, and used for the uptake assay. For these experiments, labeled EVs (10 µg) were incubated with MRC-5 cells seeded on a 24-well plate at 37 °C for 3 h. The cells were washed twice with PBS and fixed with 4% paraformaldehyde PBS at room temperature for 30 min. Cell nuclei were stained with 4′,6-diamidino-2-phenylindole (DAPI, Sigma). Images were acquired using a Nikon Eclipse Ti-S inverted microscope (Nikon, Japan) with NIS-Elements microscopy imaging software.

### qRT-PCR

Total RNA was extracted using Trizol reagent (15,596,026, Invitrogen, USA). The SeraMir exosome RNA Purification Kit (RA806A-1, System Biosciences, USA) was employed for miRNA analysis. cDNA of miRNA was synthesized following the protocol of the TaqMan microRNA assay kit (4,366,596, Thermo Fisher Scientific, Shanghai, China). qRT-PCR reactions were set up using the FastStart Universal SYBR Green Master Mix (4,309,155, Thermo Fisher Scientific, Shanghai, China). For mRNA detection, RNA was reverse transcribed into cDNA using the PrimeScript RT reagent Kit (RR047A, Takara, Japan). The synthesized cDNA was subjected to qRT-PCR using the Fast SYBR Green PCR kit (Product Number: 11,736,059, Thermo Fisher Scientific, Shanghai). Triplicates were set up for each well. U6 or GAPDH was used as an internal control. Relative expression was calculated using the 2^−ΔΔCt^ method. Experiments were repeated three times. The primer sequences used in this study for qRT PCR are provided in Table S1.

### Western blot

Total tissue proteins were extracted using the high-efficiency RIPA lysis buffer (R0010, Solarbio), strictly following the instructions. After lysis at 4 °C for 15 min, samples were centrifuged at 12,000 g for 15 min, and the supernatant was collected. Protein concentrations of each sample were determined using a BCA assay kit (20201ES76, Yeasen Biotech Co., Ltd., Shanghai). Depending on their concentrations, proteins were separated by polyacrylamide gel electrophoresis and transferred onto a PVDF membrane (ISEQ07850; Millipore, USA) using a wet transfer method. The membrane was blocked with 5% BSA at room temperature for 1 h. It was then incubated with diluted primary rabbit antibodies: α-SMA (ab5694, Abcam, UK), Collagen I (ab260043, Abcam, UK), CD9 (ab236630, Abcam, UK), Alix (ab88388, Abcam, UK), GRP94 (ab210960, Abcam, UK), and GAPDH (ab7291, Abcam, UK) on a shaker at 4 °C overnight. After washing the membrane three times with TBST for 5 min each, it was incubated with HRP-conjugated goat anti-rabbit IgG (ab6721, diluted 1:20000) for 1 h at room temperature. The membrane was rewashed three times with TBST for 5 min each and developed using the chemiluminescence reagent. Protein bands were quantified using ImageJ 1.48u software (National Institutes of Health, USA) by comparing the grayscale values of each protein to that of the internal reference GAPDH [31]. The experiment was repeated three times.

### Fluorescent enzyme reporter analysis

The sequences of SPP1 containing miR-422a binding sites and mutated binding sites were cloned into the pGL3 luciferase reporter vector (Promega, USA), generating SPP1-WT and SPP1-MUT reporter constructs. 293T cells were co-transfected with the miR-422a to mimic or mimic NC and the aforementioned reporter vectors using Lipofectamine 3000. Forty-eight hours post-transfection, luciferase activity was measured using the Dual-Luciferase Reporter Assay System (Promega, Madison, WI, USA). Relative luciferase activity was calculated as the ratio of firefly luciferase to Renilla luciferase [32, 33]. Each experiment was conducted in triplicate.

### Statistical analysis

Statistical analyses were performed using SPSS version 21.0 (IBM SPSS Statistics, Chicago, USA). Quantitative data were represented as mean ± standard deviation. Intergroup comparisons were performed using the independent samples t-test. Comparisons between two groups were conducted using the independent samples t-test, while one-way ANOVA was used for multiple group comparisons, followed by Tukey’s post hoc test. A P-value < 0.05 was considered statistically significant.

## Results

### Four EV-miRNAs associated with smoking-related COPD occurrence have been identified

**Fig. 1:**
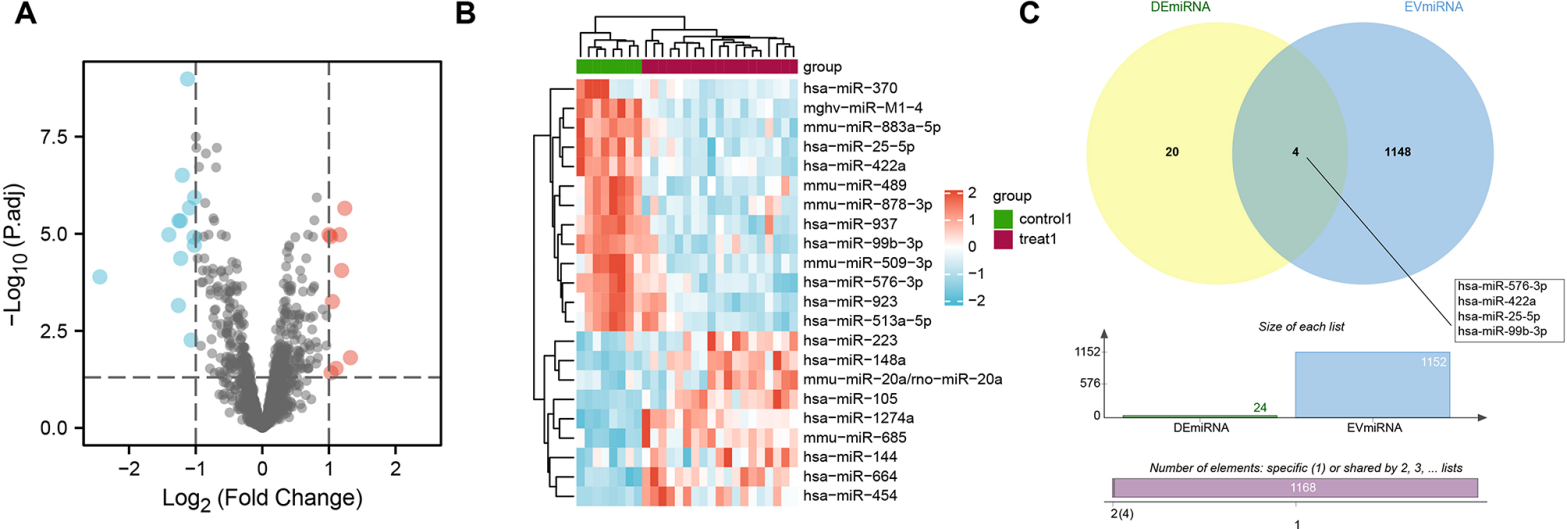
Differential gene analysis of chip GSE38974-GPL7723 and screening of candidate miRNAs associated with smoking COPD in the Vesiclepedia database. Note: (**A**) Volcano plot of miRNAs in chip GSE38974-GPL7723 (including 8 healthy controls who smoke and 19 COPD patients who smoke), with blue color indicating significantly down-regulated miRNAs and red color indicating significantly upregulated miRNAs; (**B**) Heatmap of differentially expressed miRNAs in chip GSE38974-GPL7723 (including 8 healthy controls who smoke and 19 COPD patients who smoke), with green color representing the normal group, purple color representing the disease group, red color indicating high expression, and blue color indicating low expression. The darker the color, the more significant the differential expression; (**C**) Venn diagram showing the intersection between the differential gene analysis results of chip GSE38974-GPL7723 and the search results in the Vesiclepedia database

In Fig. 1A, B, differential gene analysis was performed on-chip GSE38974-GPL7723 and 22 differentially expressed miRNAs were identified, including 9 with high expression and 13 with low expression. Intersection operations were conducted between these 22 differentially expressed miRNAs and EV-miRNAs in the Vesiclepedia database, resulting in 4 candidate miRNAs: miR-25-5p, miR-422a, miR-576-3p, and miR-99b-3p (Fig. 1C). Notably, these 4 miRNAs were all expressed at low levels in COPD samples. In conclusion, these 4 serum-derived EV-miRNAs may be associated with smoking-related COPD.

### Sixty-four mRNAs may be associated with smoking-induced COPD

**Fig. 2:**
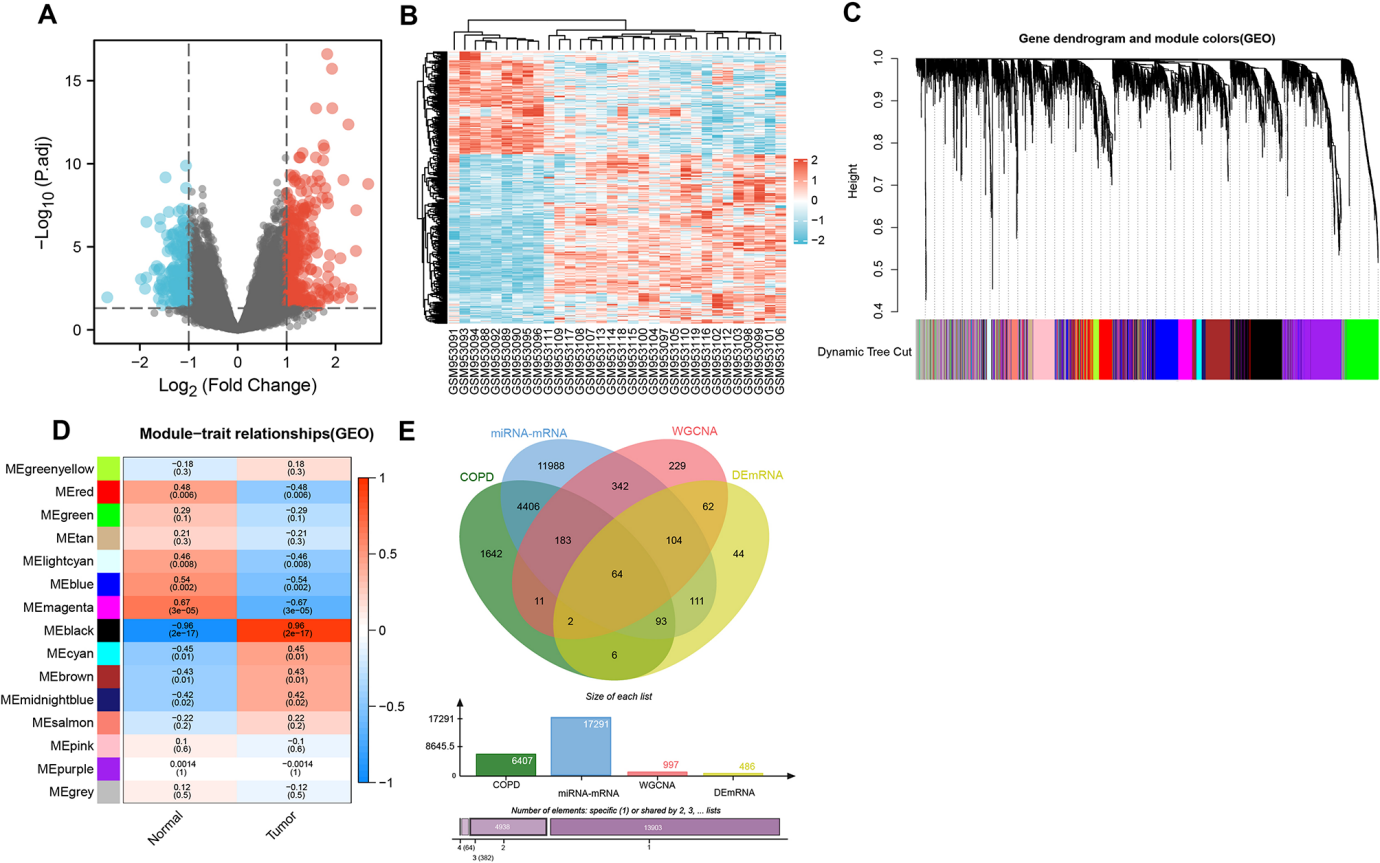
Candidate mRNAs associated with smoking COPD were filtered from the chip GSE38974-GPL4133 and online databases. Note: (**A**) Volcano plot of mRNAs in chip GSE38974-GPL4133 (9 healthy smokers and 23 smoking COPD patients samples), where blue represents significantly downregulated mRNAs and red represents significantly upregulated mRNAs; (**B**) Heatmap of differentially expressed mRNAs in chip GSE38974-GPL4133 (9 healthy smokers and 23 smoking COPD patients samples); (**C**) Cluster dendrogram of feature genes based on WGCNA analysis results in chip GSE38974-GPL4133; (**D**) Heatmap of module-trait relationships in WGCNA analysis results in chip GSE38974-GPL4133; (**E**) Venn diagram showing the intersection of downstream target genes for 4 candidate miRNAs, COPD-related genes retrieved from the GeneCards database, mRNAs included in the MEblack module, and differential gene analysis results from GSE38974-GPL4133

Further differential gene analysis was conducted on the chip GSE38974-GPL4133, identifying 486 differentially expressed mRNAs, including 302 upregulated and 184 downregulated mRNAs (Fig. 2A-B). To determine genes associated with COPD, WGCNA analysis was performed on the chip GSE38974-GPL4133, revealing 15 distinct gene modules. The MEblack module was most correlated with smoking-related COPD, thus chosen as the core module (Fig. 2C-D). Subsequently, TargetScan, miRWalk, and RNA22 databases were utilized to predict downstream target genes for four candidate miRNAs. The intersection of these downstream target genes, COPD-related genes obtained from the GeneCards database, mRNAs contained in the MEblack module, and the differential gene analysis results of GSE38974-GPL4133 led to the final selection of 64 candidate mRNAs (Fig. 2E).

### The core gene SPP1 may promote the development of smoking-induced chronic obstructive pulmonary disease (COPD) by activating the IL-17 signaling pathway

**Fig. 3:**
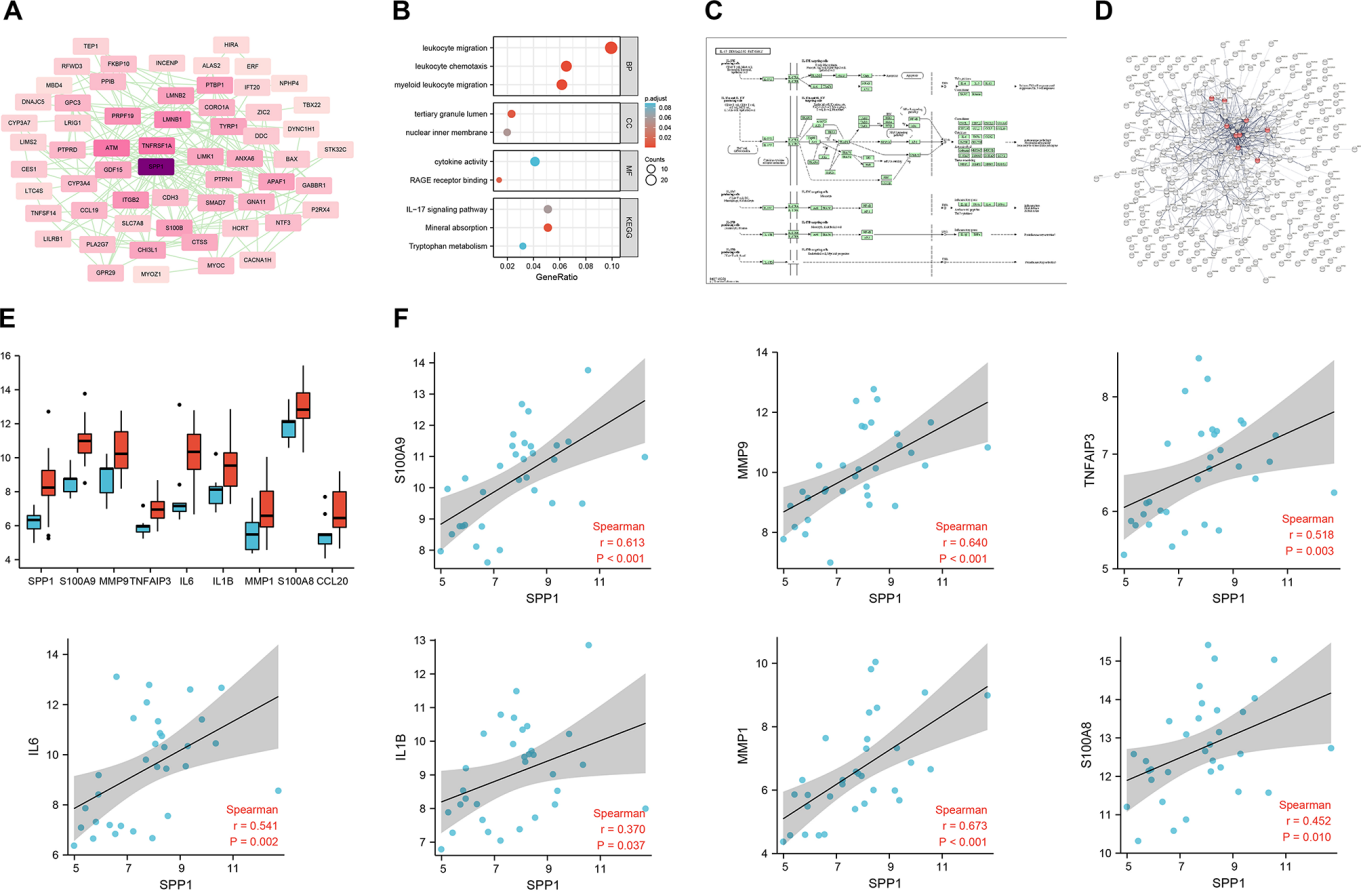
The core gene SPP1 is involved in the pathway screening of smoking-related COPD occurrence. Note: (**A**) Protein interaction network diagram of 64 candidate mRNAs, with darker colors indicating more associated molecules, with SPP1 being the darkest color; (**B**) Bubble plot of KEGG functional enrichment analysis results for SPP1-related genes, with redder colors indicating larger p-values and larger bubbles indicating more molecules included in the pathway. The x-axis represents the ratio of molecules; (**C**) The position and relationship of IL-17 A in the IL-17 signaling pathway; (**D**) the KEGG database was used to screen for genes related to SPP1 in the IL-17 signaling pathway (hsa04657); (**E**) Boxplot of the expression levels of SPP1, S100A9, MMP9, TNFAIP3, IL6, IL1B, MMP1, S100A8, and CCL20, where blue represents the normal control group, and red represents the smoking COPD group; (**F**) Correlation analysis plot of 8 genes related to SPP1 in the IL-17 signaling pathway

Next, 64 candidate mRNAs were imported into the String database, restricted to the human species, to obtain protein-protein interaction relationships. The data was then imported into the Cytoscape software to construct a PPI network. The PPI network graph consists of 60 nodes and 210 edges, with darker colors indicating higher degree values. Among the degree values, SPP1 had the highest value, suggesting that SPP1 may be the core mRNA (Fig. 3A).

Using the Pearson correlation coefficient method, correlation analysis was performed on differentially expressed mRNAs in chip GSE38974-GPL4133. 376 mRNAs correlated with SPP1 were selected based on *P* < 0.05. Further KEGG functional enrichment analysis was conducted on the SPP1-related mRNAs, and three cellular signaling pathways were identified, including the IL-17 signaling pathway, mineral absorption pathway, and tryptophan metabolism pathway (Fig. 3B).

Studies have reported that SPP1 can positively regulate the secretion of IL-17 A in lung tissue [34]. IL-17 A is important in the IL-17 signaling pathway (hsa04657) (Fig. 3C). In the IL-17 signaling pathway, there are 8 genes associated with SPP1 (Fig. 3D), namely S100A9, MMP9, TNFAIP3, IL6, IL1B, MMP1, S100A8, and CCL20. The results of the box plot (Fig. 3E) and correlation scatter plot (Fig. 3F) for the expression of these 8 genes associated with SPP1 demonstrate that all of these genes are positively correlated with SPP1 (correlation coefficient *r* > 0 and *p* < 0.05).

In summary, the above results indicate that the core gene SPP1 may promote the occurrence of smoking-induced COPD by activating the IL-17 signaling pathway.

### SPP1 functions as a downstream target of miR-422a in COPD

**Fig. 4:**
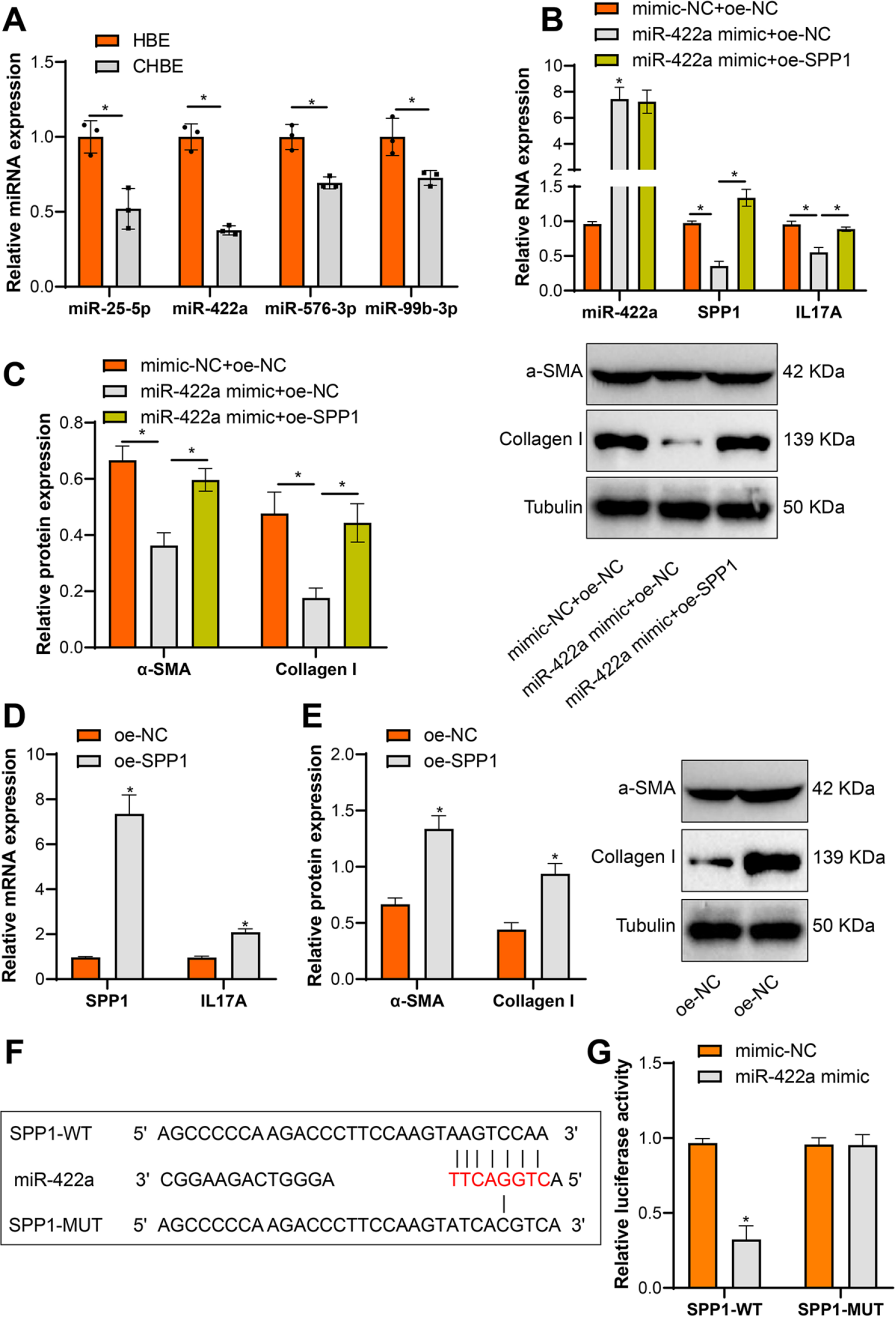
SPP1 is a downstream target of miR-422a. Note: (**A**) RT-qPCR examined the expression levels of miR-25-5p, miR-422a, miR-576-3p, and miR-99b-3p in normal HBE cells and CSE-treated HBE cells. (**B**) qRT-PCR analyzed the expression of miR-422a, SPP1, and IL17A in myofibroblasts. (**C**) Western blots determined the relative protein levels of α-SMA and type I collagen in myofibroblasts. (**D**) qRT-PCR measured the expression of SPP1 and IL17A in myofibroblasts. (**E**) Western blots assessed the relative protein levels of α-SMA and type I collagen in myofibroblasts. (**F**) SPP1, a downstream target of miR-422a, was predicted using the bibiserv-rnahybrid database (https://bibiserv.cebitec.uni-bielefeld.de/rnahybrid/). (**G**) Transfection of miR-422a mimic (or mimic NC) and SPP1-WT (or SPP1-MUT) into 293T cells was performed, and luciferase activity was analyzed using the dual-luciferase reporter gene assay after 48 h of transfection. The experiment was repeated at least three times. * indicates *P* < 0.05

In order to further identify the miRNAs involved in COPD, we examined the expression levels of four miRNAs in normal HBE cells and HBE cells treated with CSE. The results indicated that compared to the HBE group, the expression of miR-25-5p, miR-422a, miR-576-3p, and miR-99b-3p was significantly decreased in the CHBE group, with miR-422a showing the lowest expression (Fig. 4A). Therefore, miR-422a was chosen as the focus of our study. In order to validate whether miR-422a directly regulates SPP1, we further performed qRT-PCR analysis to confirm the specific regulatory effect of miR-422a on SPP1 expression. The results demonstrated an increase in miR-422a expression and a decrease in SPP1 and IL17A expression in the miR-422a mimic group compared to the mimic-NC group (Fig. 4B), along with an increase in the expression of type I collagen and α-SMA proteins (Fig. 4C). Conversely, overexpression of SPP1 (oe-SPP1) significantly increased the levels of SPP1 and IL17A in the group compared to the oe-NC group, as well as the expression of type I collagen and α-SMA proteins (Fig. 4D-E). Furthermore, by searching the bibiserv-rnahybrid database, it was found that miR-422a has target binding sites in SPP1 (Fig. 4F). To validate whether miR-422a reduces its expression by binding to the 3’UTR site of SPP1, dual-luciferase reporter assays were conducted to assess the binding capacity. The results showed that the overexpression of miR-422a inhibited the luciferase activity of WT-SPP1, while it had no effect on the activity of MUT-SPP1 (Fig. 4G). These data suggest that miR-422a directly targets and inhibits the expression of SPP1 to suppress fibroblast differentiation.

### Upregulation of extracellular vesicle miR-422a derived from HBE cells with elevated CSE treatment inhibits the myofibroblast differentiation phenotype

Previous research has reported that the accumulation of myofibroblasts may lead to changes in the structure of the lungs and other organs [35]. In addition, existing research has shown that extracellular vesicles from bronchial epithelial cells can improve the occurrence of COPD [36]. Therefore, we further investigated how bronchial epithelial cells suppress myofibroblast differentiation phenotype under CSE stress by altering the content of miR-422a in their EVs.

**Fig. 5:**
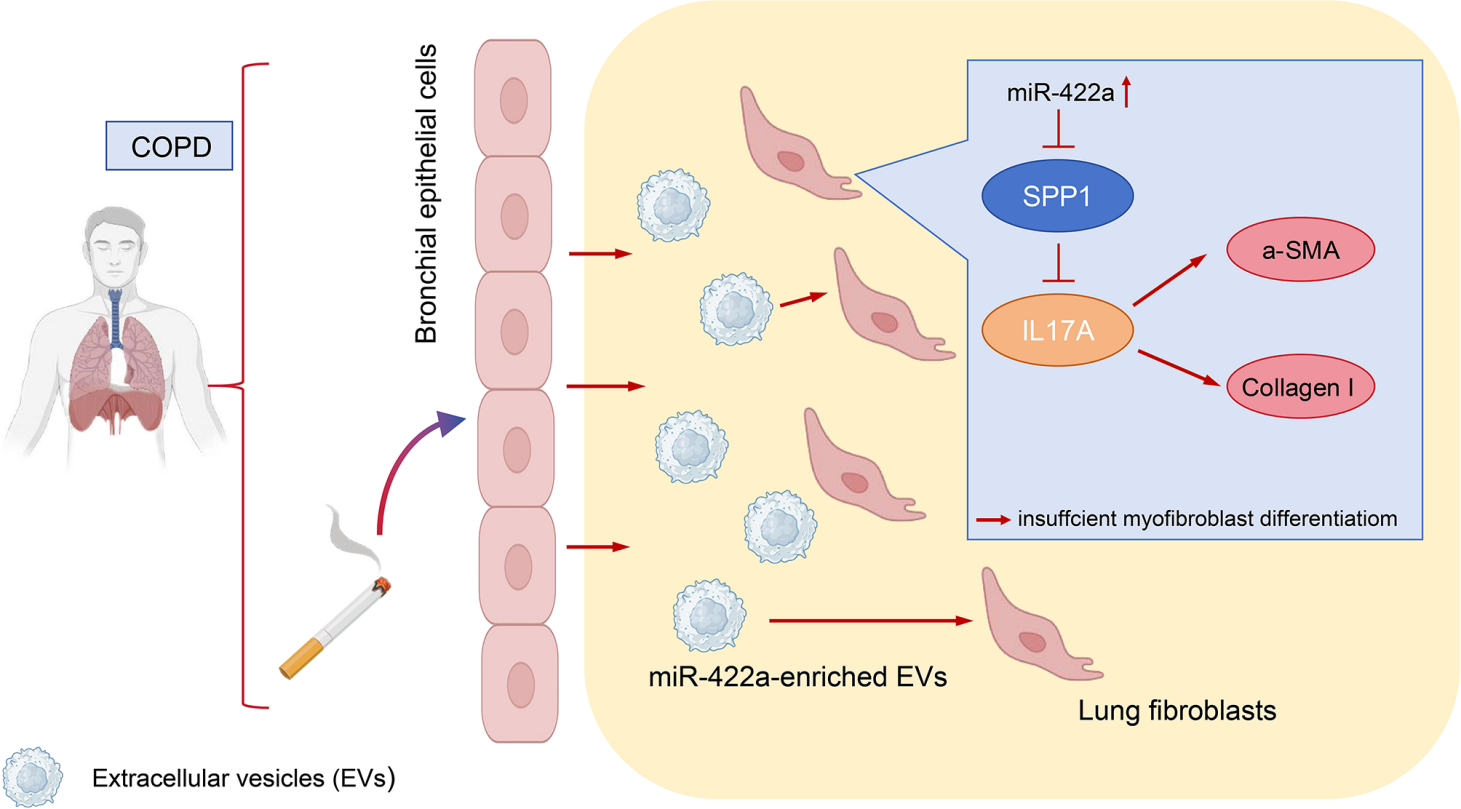
Extracellular vesicles from HBE cells transfer miR-422a to MRC-5 cells, regulating myofibroblast differentiation phenotype. Note: ExoQuick-TC was used to isolate extracellular vesicles from normal or CSE-treated HBE cells. (**A**) Transmission electron microscopy images of extracellular vesicles (bar = 100 nm); (**B**) Particle number and size of HBE-exo or CHBE-exo were measured using the ZetaView® nanoparticle tracking analyzer based on dynamic light scattering; (**C**) Expression of CD9, Alix, and GRP94 in extracellular vesicles was examined by Western blot; (**D**) The levels of miR-422a in extracellular vesicles from normal or CSE-treated HBE cells were detected by qRT-PCR. (**E**) Microscopic images of MRC-5 cells incubated with PKH67-labeled (green) extracellular vesicles, with cell nuclei stained with DAPI (blue); (**F**) Co-culture of MRC-5 cells with extracellular vesicles (50 µg/mL) from normal or CSE-treated HBE cells, with expression levels of miR-422a, SPP1, and IL17A in MRC-5 cells detected by qRT-PCR; (**G**) Relative protein levels of α-SMA and type I collagen in MRC-5 cells were measured by Western blot. HBE-EVs, extracellular vesicles derived from normal HBE cells; CHBE-EVs, extracellular vesicles derived from CSE-treated HBE cells. Experiments were repeated at least three times. * indicates *P* < 0.05

Nanovesicles with a diameter of ≤ 200 nm were found in extracellular vesicles isolated from HBE cells treated with normal and CSE processing, consistent with the characteristic size range of extracellular vesicles (Fig. 5A). Using the ZetaView® Nanoparticle Tracking Analyzer, no significant differences were observed in particle size and count between extracellular vesicles derived from normal and CSE-treated HBE cells (Fig. 5B). Immunoblotting characterization and quantification of HBE-EVs and CHBE-EVs revealed the expression of extracellular vesicle markers CD9 and Alix but not the endoplasmic reticulum marker GRP94 (Fig. 5C).

In addition, extracellular vesicles from normal and CSE-treated HBE cells were added to MRC-5 cells. After 24 h, uptake of the extracellular vesicles was observed, indicating internalization of PKH67-labeled extracellular vesicles (Fig. 5D). qRT-PCR analysis showed that the level of miR-422a in extracellular vesicles from CSE-treated HBE cells was lower than that in normal HBE cells. To demonstrate the function of miR-422a, we upregulated the level of miR-422a in HBE cells. The results showed a significant increase in the expression level of miR-422a in the CHEB-EVs miR-422a group compared to the CHEB-EVs miR-NC group (Fig. 5E).

Further co-culturing EVs with MRC-5 cells, the miR-422a level in MRC-5 cells decreased in the CHEB-EVs group compared to the HEB-EVs group, while the mRNA expression of SPP1 and IL17A, as well as the protein levels of α-SMA and collagen I, increased. Compared to the CHEB-EVs miR-NC group, the miR-422a level in MRC-5 cells significantly increased in the CHEB-EVs miR-422a group, while the mRNA expression of SPP1 and IL17A, as well as the protein levels of α-SMA and collagen I, decreased (Fig. 5F-G).

In summary, upregulation of extracellular vesicle miR-422a derived from HBE cells undergoing CSE treatment can target and inhibit the expression of SPP1, thereby suppressing myofibroblast differentiation phenotype.

## Discussion

Chronic obstructive pulmonary disease (COPD) remains a formidable health challenge globally, consistently demonstrating escalating incidence and mortality rates [37]. Traditionally, the focus of COPD research has predominantly been on its inflammatory response and airway remodeling. In terms of the inflammatory response, bronchial epithelial cells excessively produce various triggering factors. As a source of various cytokines/chemokines, adhesion molecules, and growth factors, bronchial epithelial cells regulate the response of other components of the airway wall and immune cells against cigarette smoke [38]. Regarding airway remodeling, bronchial epithelial cells play a crucial role in fine-tuning the balance between epithelial cells and mesenchymal cells [39, 40]. It is evident that bronchial epithelial cells play a critical role in regulating the pathology and progression of COPD.

Recent strides in the field have identified extracellular vesicles (EVs) as quintessential modulators of cell-to-cell communication, invoking immense research interest given their pivotal roles across diverse physiological and pathological landscapes [41]. In recent years, extracellular vesicles (EVs) have gained significant interest as key mediators of intercellular communication in various physiological and pathological processes [41]. Particularly, the miRNA-mRNA regulatory network has been implicated in multiple diseases. Although studies have indicated that EVs derived from smoking-induced bronchial epithelial cells can promote differentiation and inhibit autophagy in lung fibroblasts by upregulating miR-210 and targeting ATG7 [42], the impact of relevant miRNAs in EVs secreted by bronchial epithelial cells on the pathophysiology of COPD remains poorly understood. Therefore, investigating the complex signaling regulation of COPD pathological tissue remodeling induced by EV-carried miRNAs is crucial for a deeper understanding of these mechanisms and for providing new strategies for disease prevention and early treatment [43].

While prior research has emphasized miR-422a’s prominence in diverse conditions, particularly in oncological and cardiovascular domains [44, 45], our meticulous analysis, anchored on the GEO database, has unveiled miR-422a’s instrumental role in tobacco-aggravated COPD. Moreover, we have innovatively identified SPP1 as a downstream target gene of miR-422a, which regulates the involvement of IL-17 A in fibroblast differentiation. This finding provides new evidence and understanding of the role of miR-422a in respiratory system diseases. Furthermore, we have explored the roles of SPP1 and IL-17 A in COPD. Earlier studies have confirmed their critical roles in COPD, but their exact mechanisms remain unclear [46–48]. Our research has revealed their novel roles in the regulatory network of miR-422a, thus opening up new avenues for investigating the roles of SPP1 and IL-17 A in COPD. Additionally, contrary to the previous belief that EVs merely act as biomarkers in COPD, our study provides fresh evidence and an in-depth understanding of the involvement of EVs in the pathological process of COPD.

However, we must acknowledge that the miRNA-mRNA regulatory network mediated by EVs is an exceedingly complex mechanism. Due to the fact that EVs contain cell surface proteins almost identical to their source cells, they can fuse with target recipient cells [49]. When these EVs are taken up by recipient cells, they transfer various biomolecules, including miRNA, thereby altering the biological activity of the recipient cells and affecting the pulmonary microenvironment [50, 51]. In our screening conditions, we identified four differential miRNAs: miR-25-5p, miR-576-3p, miR-99b-3p, and miR-422a. Regarding miR-25-5p, miR-25-5p in EVs modified by Nell inhibits osteogenesis of bone marrow mesenchymal stem cells (BMSCs) through targeting Smad2 and inhibiting the SMAD and extracellular signal-related kinase 1 and 2 (ERK1/2) pathways [52]. M2 macrophages activate autophagy in foot cells of diabetic nephropathy (DN) induced by high glucose through the secretion of exosomal miR-25-3p, which suppresses the expression of DUSP1 [53]. In the case of miR-576-3p within EVs, there is no mechanistic study, only literature reports as a biological marker and diagnostic indicator for breast cancer brain metastasis and triple-negative breast cancer (TNBC) [54, 55]. Concerning miR-99b-3p, mesenchymal stem cell-derived EVs (MSC-EVs) promote autophagy of microglial cells through the transfer of miR-99b-3p, thereby relieving neuropathic pain [56]. It may also have an anti-apoptotic/anti-cell death effect in radiation-induced or intraperitoneal disease [57, 58]. Regarding miR-422a, plasma exosomal miR-422a serves as the optimal diagnostic biomarker for ischemic stroke [59]. Moreover, miR-422a mediates the intercellular interaction and targets PLP2 to promote ovarian cancer (OC) development within exosomes [60]. It is evident that none of these four differential miRNAs have been studied in the field of COPD, suggesting significant research potential. However, the selection of miR-422a as the research subject due to its lowest expression level seems to have limitations, which undermines the broad applicability of this study. Alternatively, an integrated analysis of the four differential miRNAs would be more convincing, but this requires further implementation in future studies. Furthermore, when conducting further KEGG pathway enrichment analysis on SPP1-related mRNAs, three cell signaling pathways were identified: the IL-17 signaling pathway, mineral absorption pathway, and tryptophan metabolism pathway. Currently, there is no research on mineral absorption in tracheal and lung tissue diseases, presenting a significant challenge as a research subject. Conversely, tryptophan metabolites are emerging as novel metabolic biomarkers that distinguish male COPD patients from control groups [61], which contrasts with our previous bioinformatics analysis. Additionally, it has been demonstrated that tryptophan metabolism increases in HIV, but it does not appear to independently cause HIV-COPD, which differs from the etiology of smoking-induced COPD studied in our research [62]. While metabolomics has indeed provided new insights into the prediction and treatment of various diseases, whether SPP1 is involved in the tryptophan metabolism pathway remains unconfirmed, necessitating further analysis in conjunction with COPD metabolomics data in the future. The fact that SPP1 positively regulates the IL-17 signaling pathway [34] supports our results and provides a research direction for this study. Perhaps the combination of the aforementioned four differential miRNAs, mineral absorption pathway, and tryptophan metabolism pathway also represent functionally significant signaling pathways in COPD.

Chronic obstructive pulmonary disease (COPD), aside from being induced by exposure to factors such as smoking, is frequently associated with acute exacerbations that are linked to bacterial, viral, and eosinophilic inflammation (DOI: 10.1164/rccm.201104-0597OC). The prediction and treatment of these malignant exacerbations are currently limited. It remains unclear whether the information carried by extracellular vesicles (EVs) is involved in the complex interactions between inflammation and microbial processes. Additionally, the specific mechanisms and potential negative impacts of COVID-19 on COPD are yet to be fully understood; however, it is confirmed that COPD patients are at a higher risk for the infection. Moreover, there is an increased incidence of cardiovascular diseases in COPD patients compared to the general population, and aging, smoking, environmental pollutants, gender, and diet are common influencing factors for COPD and cardiovascular disease. Furthermore, the symptoms of pulmonary heart disease caused by lung diseases are consistent, often leading to the neglect of one disease in patients with another condition [63]. These findings suggest that COPD can be influenced by multiple factors, apart from early smoking induction, and the degree of involvement of EVs in this process warrants further investigation.

The sources of EVs obtained in this study have certain limitations. Previous studies have indicated that EVs can be released by various cells in the airways, including bronchial epithelial cells, endothelial cells, alveolar macrophages, and other immune cells [4]. Additionally, bronchoalveolar lavage fluid (BALF)-derived EVs, containing DNA and utilized for epidermal growth factor receptor (EGFR) gene typing, exhibit higher consistency in traditional tissue biopsies compared to plasma EVs, with a sensitivity of up to 100% [64]. As for miRNA, which is currently being extensively studied as a biomarker, it is associated not only with EVs in the bloodstream but also with proteins such as Argonaute complexes [65] or lipoprotein particles [66]. Furthermore, since the available isolation methods cannot guarantee the complete purification of EVs from these contaminants, the use of EVs from non-lipoprotein and pure protein bioliquids, such as BALF, might serve as a better source for EV-related miRNA biomarkers. Therefore, the study of EVs obtained from bronchoalveolar lavage fluid (BALF) appears to provide a more comprehensive insight into the pathology of COPD, despite being one of the limitations of this study and a potential avenue for future research.

In conclusion, this study offers a novel perspective, thoroughly investigating the associations and roles of EVs, miR-422a, SPP1, and IL-17 A in early-stage COPD. Although there are limitations, it provides valuable directions and insights for future COPD research. It is hoped that future studies can further deepen these findings and provide more effective strategies for the prevention and treatment of COPD.

## Conclusions

**Fig. 6:**
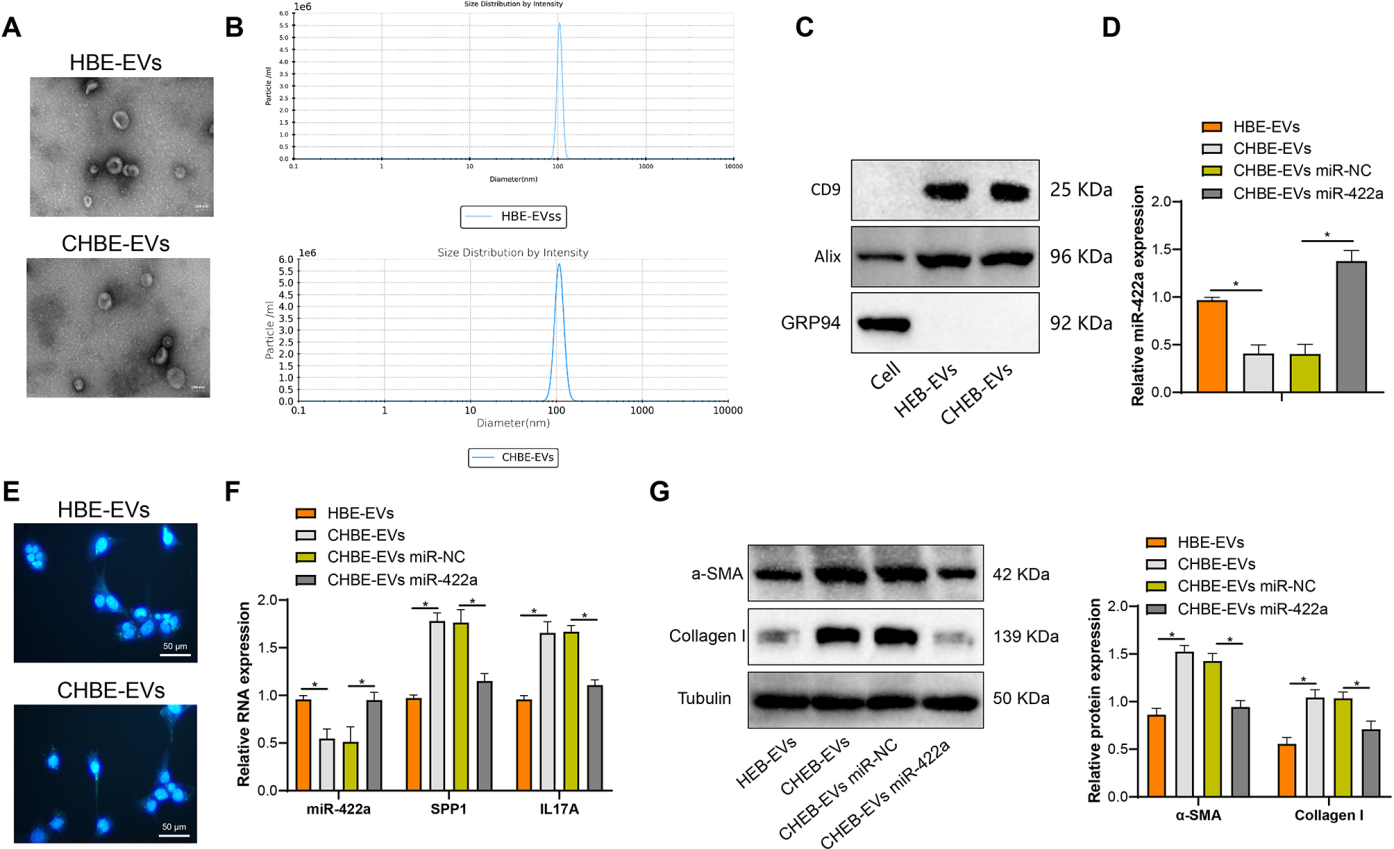
Schematic representation of the molecular mechanism underlying the delayed occurrence of chronic obstructive pulmonary disease in smokers through bronchial epithelial cells transmitting miR-422a-targeted inhibition of SPP1 in myofibroblasts via extracellular vesicles (EVs)

To encapsulate, our investigation breathes fresh life into the discourse, meticulously probing the interrelationships and contributions of EVs, miR-422a, SPP1, and IL-17 A to the COPD narrative (Fig. 6). Although our study isn’t devoid of limitations, it undoubtedly paves the way for ensuing research endeavors. It is our fervent aspiration that future explorations will enrich these insights, spawning novel, efficacious strategies for COPD prevention and management.

### Electronic supplementary material

Below is the link to the electronic supplementary material.

Supplementary Material 1

Supplementary Material 2

## Data Availability

The datasets generated and/or analyzed during the current study are available from the corresponding author on reasonable request.

## Abbreviations

COPD: Chronic Obstructive Pulmonary Disease
EVs: extracellular vesicles
PPI: Protein-Protein Interaction
AHR: Airway hyperreactivity
CSE: Cigarette Smoke Extract
MEM: Minimum Essential Medium
HBE: Human Bronchial Epithelial
