# Supplementary material for: Extracellular vesicle dynamics in COPD: understanding the role of miR-422a, SPP1 and IL-17 A in smoking-related pathology
Source: BMC Pulm Med. 2024 Apr 12;24:173. doi: 10.1186/s12890-024-02978-y (PMC11010439; doi:10.1186/s12890-024-02978-y)
Supplement: Supplementary file 1 — Supplementary Material 1 [file 12890_2024_2978_MOESM1_ESM.docx]

**Table S1** The primer sequences for qRT-PCR

| Gene | Sequence |
| --- | --- |

| miR-422a | Forward: ACTGGACTTAGGGTCAGAAGGC |
| --- | --- |

|  | Reverse: universal primer |
| --- | --- |
| U6 | Forward: GCTTCGGCAGCACATATACTAA |
|  | Reverse: universal primer |
| SPP1 | Forward: CTCCATTGACTCGAACGACTC |
|  | Reverse: CAGGTCTGCGAAACTTCTTAGAT |
| IL17A | Forward: AGATTACTACAACCGATCCACCT |
|  | Reverse: GGGGACAGAGTTCATGTGGTA |
| GAPDH | Forward: AAGCCCATCACCATCTTCCAGGAG |
|  | Reverse: AGCCCTTCCACAATGCCAAAG |
