# Supplementary material for: Extracellular vesicle dynamics in COPD: understanding the role of miR-422a, SPP1 and IL-17 A in smoking-related pathology
Source: BMC Pulm Med. 2024 Apr 12;24:173. doi: 10.1186/s12890-024-02978-y (PMC11010439; doi:10.1186/s12890-024-02978-y)
Supplement: Supplementary file 2 — Supplementary Material 2 [file 12890_2024_2978_MOESM2_ESM.docx]

**Original WB Gels image file**


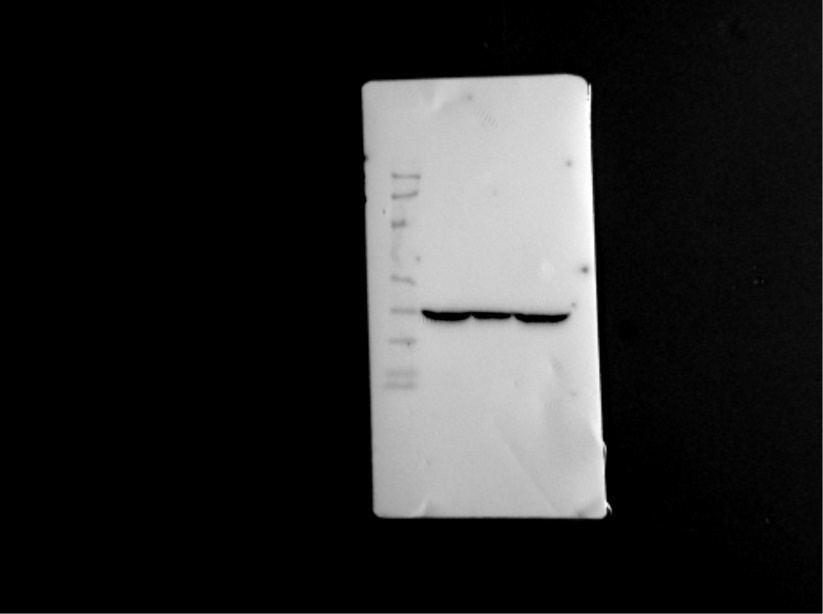


Original images of Original images of Figure4C-1


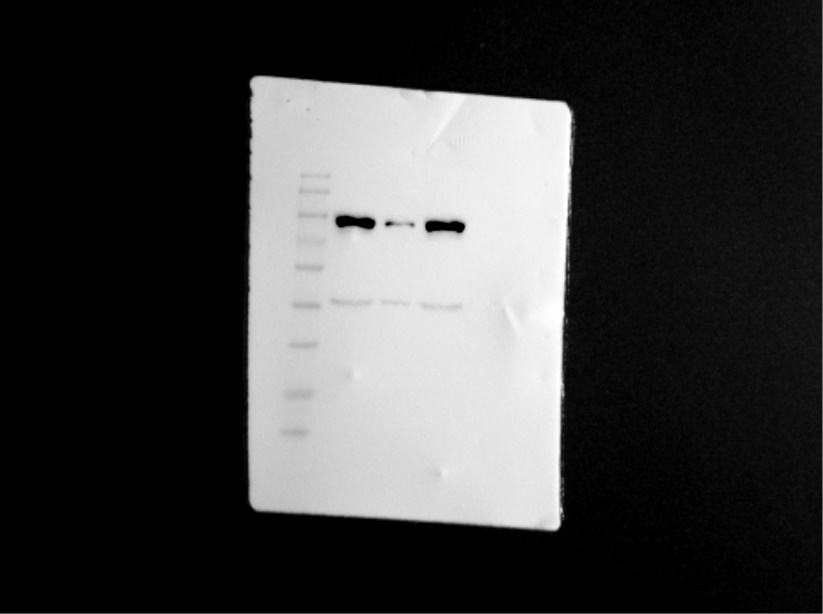


Original images of Figure4C-2


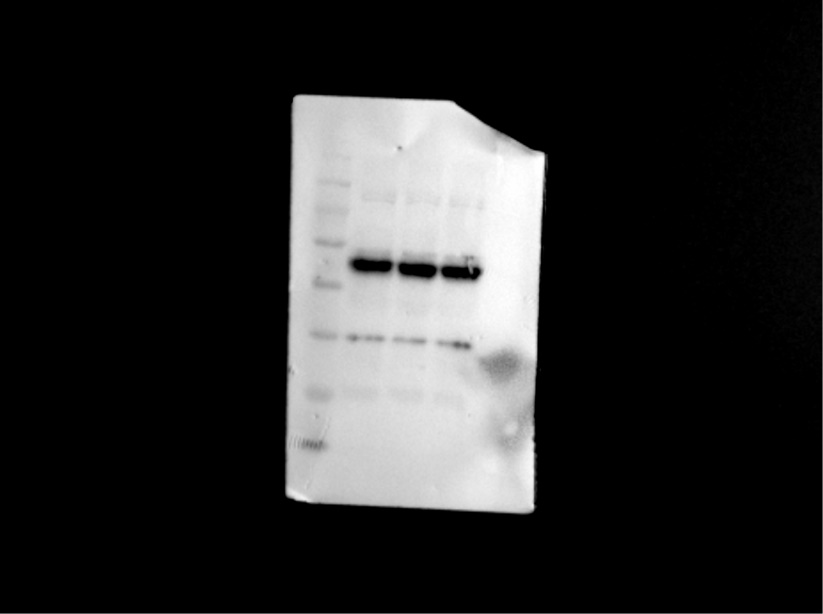


Original images of Figure4C-3


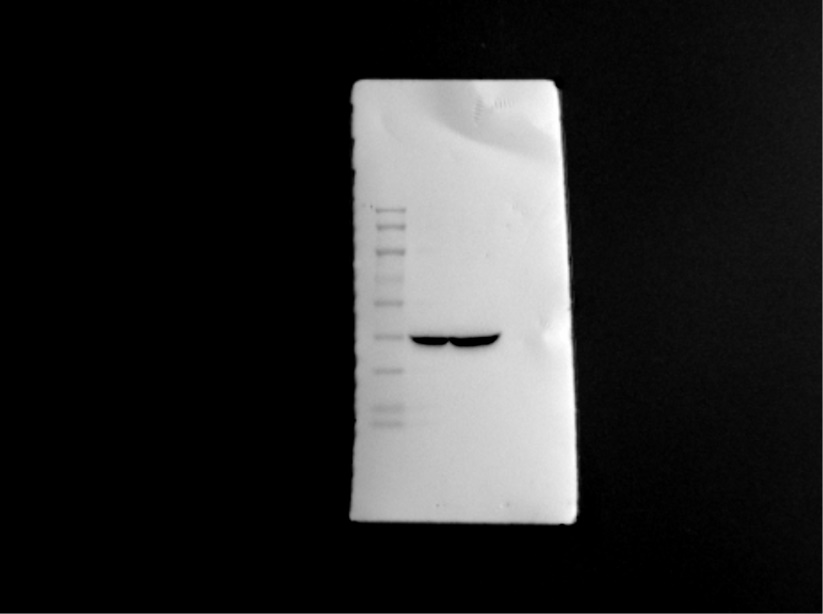


Original images of Figure4E-1


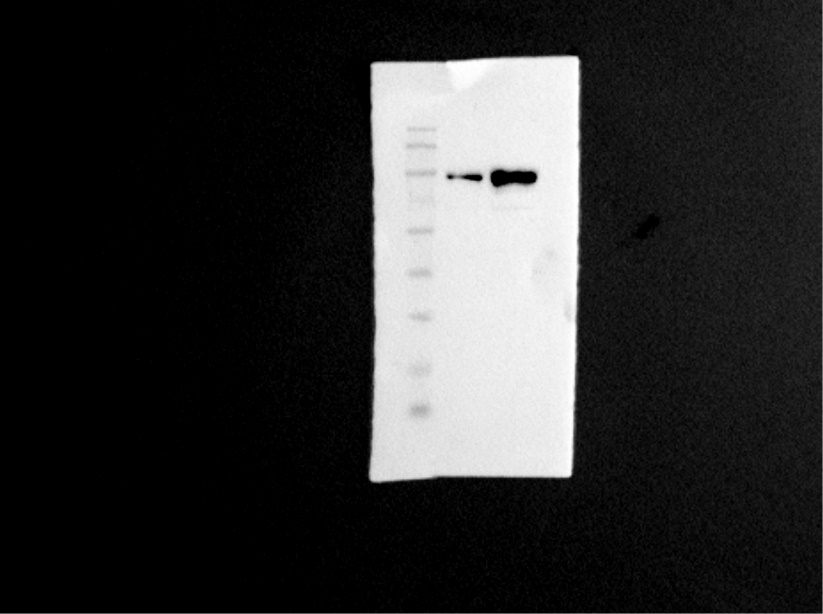


Original images of Figure4E-2


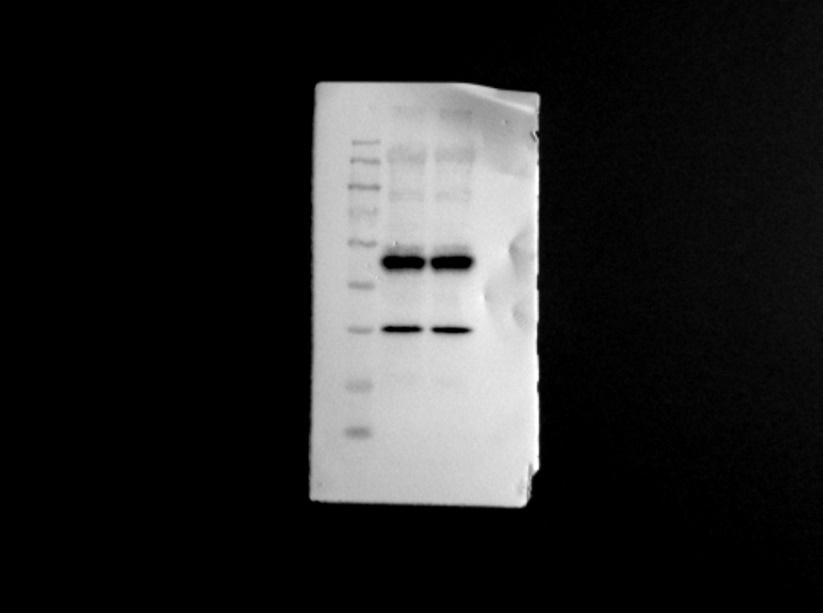


Original images of Figure4E-3


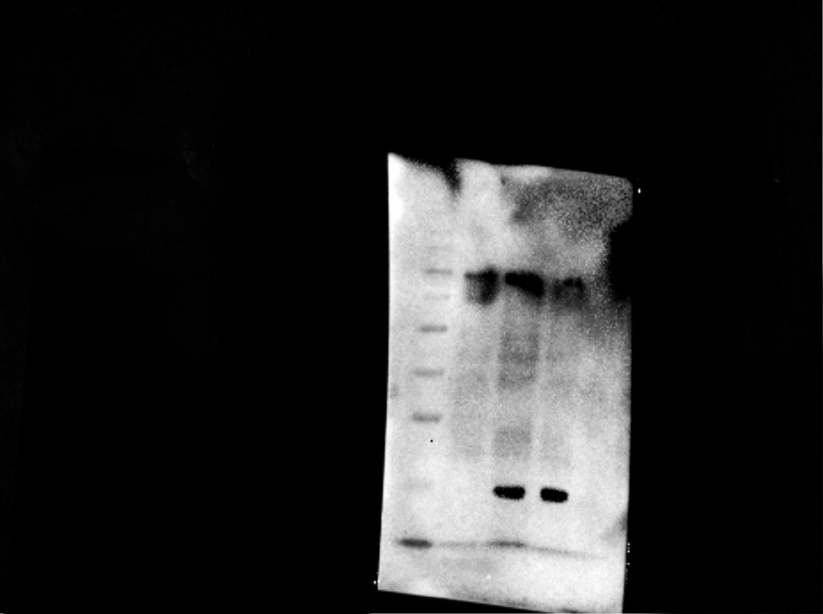


Original images of Figure5C-1


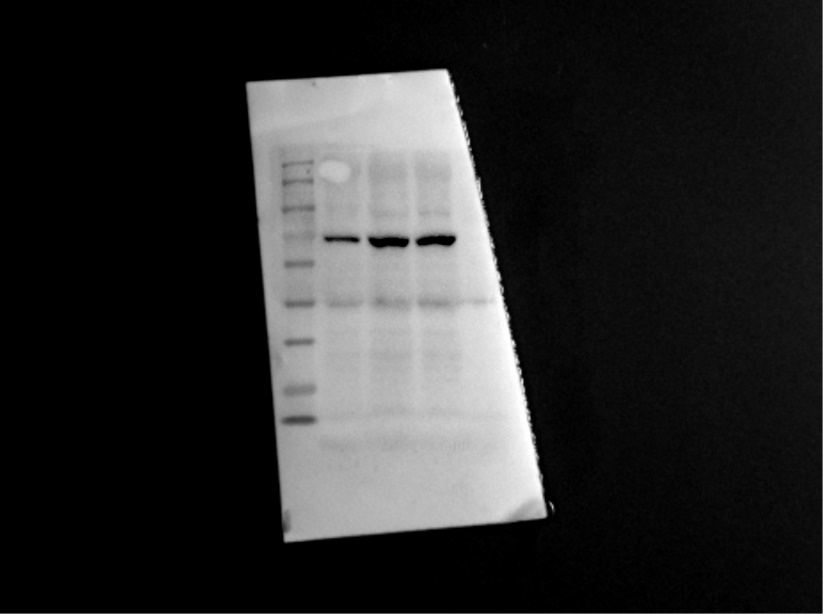


Original images of Figure5C-2


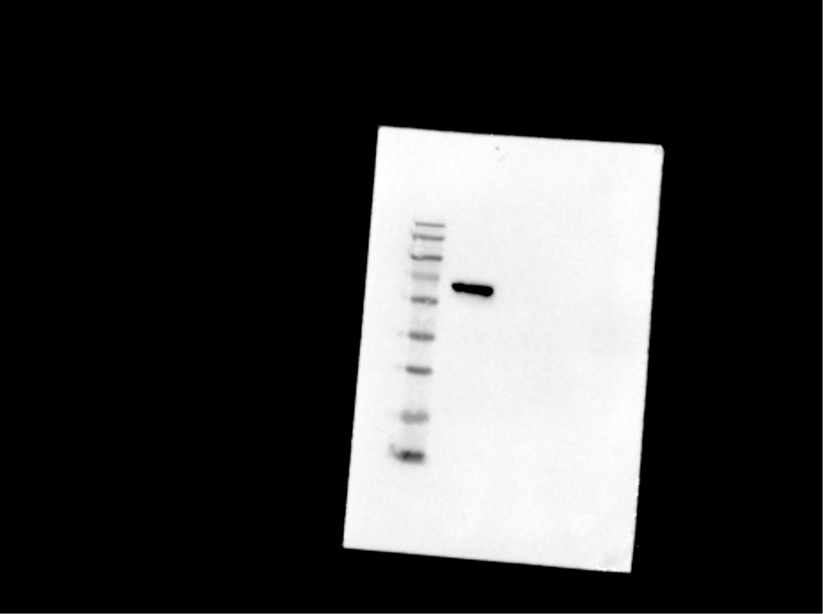


Original images of Figure5C-3


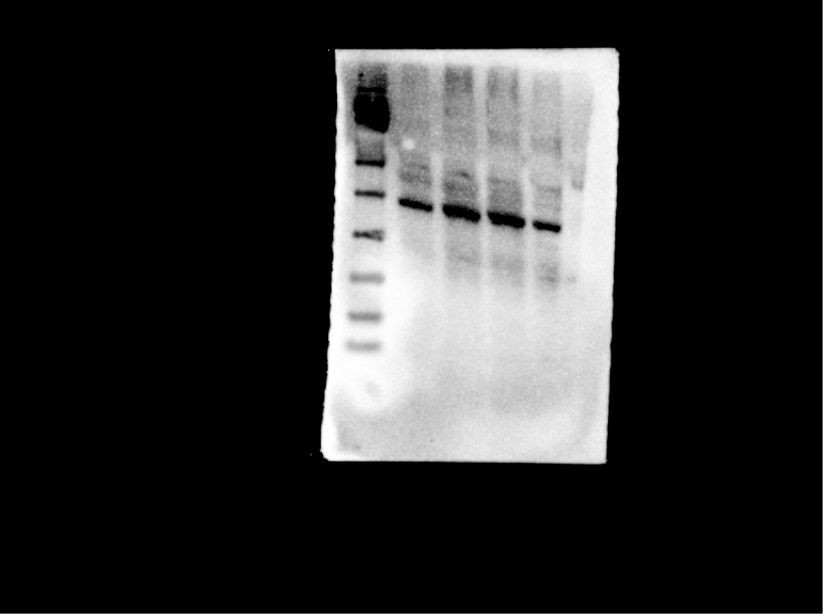


Original images of Figure5G-1


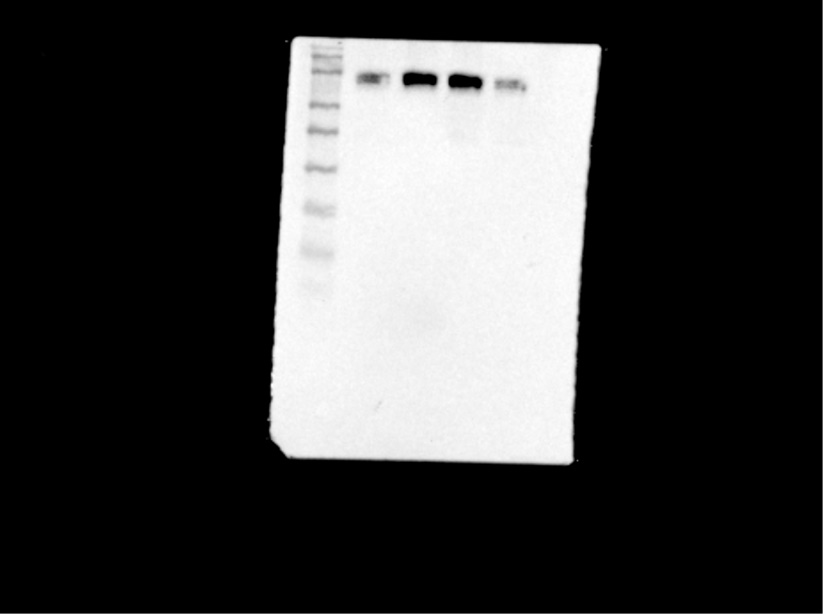


Original images of Figure5G-2


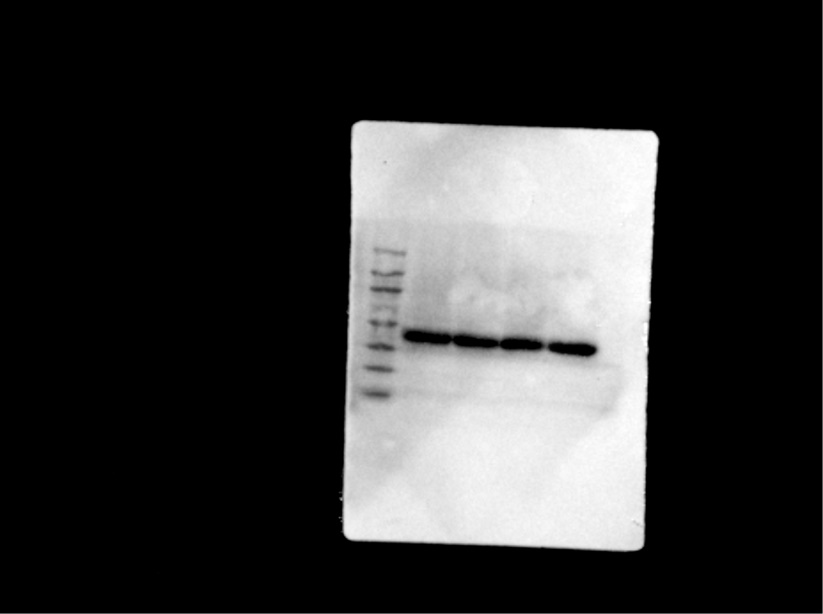


Original images of Figure5G-3
